# Should transcutaneous bilirubin be measured in preterm infants receiving phototherapy? The relationship between transcutaneous and total serum bilirubin in preterm infants with and without phototherapy

**DOI:** 10.1371/journal.pone.0218131

**Published:** 2019-06-14

**Authors:** Christian V. Hulzebos, Deirdre E. Vader-van Imhoff, Arend F. Bos, Peter H. Dijk

**Affiliations:** Department of Pediatrics, Division of Neonatology, Beatrix Children’s Hospital, University Medical Center Groningen, Groningen, the Netherlands; Centre Hospitalier Universitaire Vaudois, FRANCE

## Abstract

Our objective was to analyze the relationship between transcutaneous bilirubin (TcB) measured on an unexposed area of skin and total serum bilirubin (TSB) in preterm infants before, during, and after phototherapy (PT). For this purpose paired TSB and TcB levels were measured daily during the first ten days after birth in preterm infants of less than 32 weeks’ gestation. TcB was measured with a Dräger Jaundice Meter JM-103 on the covered hipbone. Agreement between TSB and TcB levels was assessed before, during, and after PT. True negative and corresponding false negative percentages were calculated using different TcB cut-off levels. Data are presented as mean (±SD). We obtained 856 paired TcB and TSB levels in 109 preterm infants (66 boys, gestational age 29.4 ± 1.6 weeks and birth weight 1282 g ± 316 g). We found that the difference between TSB and TcB before PT was significantly lower, 44 (±36) μmol/L, than the difference during and after PT, 61 (±29) μmol/L and 63 (±25) μmol/L, respectively; *P* < 0.01. Blood sampling could be reduced by 42%, with 2% false negatives, when 50 μmol/L was added to the TcB level at 70% of the PT threshold. Our conclusion is that phototherapy enhances underestimation of TSB by TcB in preterms, even if measured on unexposed skin. The use of specific TcB cut-off levels substantially reduces the need for TSB measurements.

## Introduction

Transcutaneous bilirubin (TcB) levels provide a quick estimate of TSB levels based on the spectrophotometric measurement of the yellow color of the skin and subcutaneous tissue. As early as 1980, Yamanouchi and colleagues predicted the potential screening value of TcB [1]. Currently, TcB measurements are advocated for the purpose of screening unconjugated hyperbilirubinemia in infants with a gestational age (GA) of more than 35 weeks [2]. This measure has also proven to be reliable in preterm infants of less than 35 weeks’ gestation [3] and specific TcB cut-off thresholds were published for infants between 28–34 + 6 weeks to “identify infants that require a total serum bilirubin (TSB) to confirm or exclude the need for phototherapy (PT)” [4].

TcB measurements are not routinely performed during (PT). The accuracy of TcB in infants receiving PT is lower due to skin bleaching [5,6]. Nevertheless, the vast majority of early preterm infants receive PT [7,8]. As a logical consequence, frequent blood sampling is necessary to measure infants’ TSB levels and to assess treatment efficacy in order to manage hyperbilirubinemia adequately. Frequent blood sampling is associated with risks, such as pain, infection, and anemia. Thus the question whether TcB should be used to assess the efficacy of PT in preterm infants, in addition to screening is highly relevant. To overcome TcB inaccuracy during PT, a photo-opaque patch attached to the infant’s forehead or sternum may be used to shield the skin from the PT light. Data on TcB levels during PT measured on covered skin sites show a higher correlation with TSB compared to TcB levels from uncovered areas of skin [9–16]. Data in preterms on TcB–TSB correlations measured on other areas of skin during PT, for instance, the hipbone covered by a diaper, are not available. To rely solely on TcB measurements means that high TSB levels, which require treatment, may not be missed. Yet, it is well-known that TcB tends to underestimate TSB levels, especially at higher levels [17]. Various formulas have been developed to determine TcB cut-off levels that minimize the risk of missing high TSB levels, i.e. cut-off levels that produce only a few false negative (FN) TcB measurements [4,18,19]. Nevertheless, data on this issue in early preterm infants are scarce.

Our first aim was to assess the influence of PT on TcB levels. We analyzed the relationship between TcB and TSB levels in preterm infants of less than 32 weeks’ gestation before, during, and after phototherapy using TcB measurements taken on the covered hipbone. Secondly, we searched for TcB cut-off levels that would reduce the frequency of blood sampling with a minimal risk of missing high TSB levels in this particular group of preterm infants.

## Materials and methods

### Patients

The study was approved by the Medical Ethics Committee of University Medical Center Groningen and carried out in a subgroup of patients included in a multicenter, randomized, controlled trial investigating the bilirubin/albumin ratio in preterm infants treated for hyperbilirubinemia (bilirubin/albumin ratio trial (BARTrial), ISRCTN74465643) [20]. The subgroup consisted of infants admitted to the neonatal intensive care unit (NICU) of Beatrix Children’s Hospital, University Medical Center Groningen. The TcB measurements were taken after written informed consent had been given by the parents or guardians of all the infants. Inclusion criteria were a GA of less than 32 weeks and admittance to a Level III NICU within 24 hours after birth. Exclusion criteria of the BARTrial were major congenital malformations, clinical syndromes, or chromosomal abnormalities. The reporting guideline required for this study, the STARD checklist, is presented in S1 Table.

### Measurement of TSB and TcB levels

Prospectively, we determined TSB and TcB levels daily during the first ten days after birth. Additional TSB measurements were performed on request of the attending neonatologist, for example, if TSB rose quickly or approached the exchange transfusion threshold.

Within one hour before or after blood sampling for TSB analysis (of a total blood volume of 250–500 μL), the TcB measurement was performed with the Minolta Air Shields Jaundice Meter 103 (JM– 103, Dräger Medical, Lübeck, Germany). The JM-103 instrument determines the difference in skin reflectance between optical densities for light in the blue (450 nm) and green (550 nm) wavelength regions. By using two optical paths the reflectance of melanin, dermal maturity, and hemoglobin from the superficial tissue can be deducted. The corresponding TcB level reflects the cutaneous and subcutaneous bilirubin content corrected for GA and ethnicity [21]. All NICU nurses were instructed to measure TcB on the hipbone underneath the infant’s diaper. The JM– 103 displayed the mean TcB level of three consecutive measurements in μmol/L. Regular calibration of the bilirubinometer was ensured, following the instructions of the manufacturer.

TSB was determined in 10 μL of serum using the modified diazo method (Roche Modular, Roche Diagnostics, Almere, the Netherlands) and determined immediately. Our laboratory is connected to the Dutch Foundation for Quality Assessment in Medical Laboratories (SKML), which provides a specific quality assessment scheme for neonatal samples (for every Dutch laboratory) in order to detect abnormal variability and to improve the measurement method. Our laboratory participates in this national quality assessment program.

### Phototherapy

Phototherapy was given according to the Dutch TSB-based PT thresholds for preterm infants of less than 35 weeks’ GA with hyperbilirubinemia [22]. These TSB thresholds are set by postnatal age (in hours) and divided into five birth weight categories (< 1000 g, 1000–1249 g, 1250–1499 g, 1500–2000 g, and > 2000 g). For preterm infants with standard risk the thresholds are 100 μmol/L, 150 μmol/L, 190 μmol/L, 220 μmol/L, and 240 μmol/L, respectively [22]. All nomograms can be accessed at the following Dutch-language web address: http://babyzietgeel.nl/kinderarts/hulpmiddelen/diagnostiek/bilicurves_prematuren.php.

PT was given via conventional overhead devices with fluorescent tubes (Medela PT lamp, Medela AG Medical Technology, Baar, Switzerland or Dräger PT Unit 4000, Dräger Medical, Lübeck, Germany) and/or one fiberoptic pad with a halogen lamp (Biliblanket Plus Ohmeda, Ohmeda Medical, Columbia, Maryland, USA). LED-based PT was not used during the study period. Median irradiances varied between 8–13 μW/cm/nm and 17 μW/cm/nm for the overhead and underneath devices, respectively. PT was registered in hours of use.

### Statistics

First, the overall correlation of TSB and TcB levels was calculated using all measurements. Subsequently, we calculated the correlation between TSB and TcB levels before, during, and after PT. The mean TSB and the mean difference between TSB and TcB levels were calculated and depicted in Bland Altman plots for all levels and for levels before, during, and after PT [23].

The mean difference between TSB and TcB levels was calculated for different birth weight categories, viz. < 1000 g, 1000–1249 g, 1250–1499 g, and 1500–2000 g (not for > 2000 g due to the relatively small number of cases in this category), for different GA categories, viz. ≤ 26 weeks, 26+1 to 28 weeks, 28+1 to 30 weeks, and 30+1 to 32 weeks, and for time (h) after PT was stopped. Significant differences were determined using ANOVA. Post hoc we did a multiple comparison analysis by using the Bonferroni test. A *P* value of less than 0.05 was considered statistically significant.

The TcB cut-off levels were defined as TcB levels at which TSB measurements were indicated to assess the degree of hyperbilirubinemia and were based on the Dutch TSB thresholds of PT (ie, the PT threshold) [22]. Because of the increasing slope of the PT thresholds during the first 24 to 48 hours after birth we used the PT threshold after 48 postnatal hours to define TcB cut-off levels. We assessed four of the formulas that have been proposed in the literature with TcB cut-off levels and that would be most optimal in reducing the number of blood samples without missing high TSB levels per birth weight category. 1.) TcB cut-off levels equivalent to the PT thresholds and using the measured TcB, 2.) TcB cut-off levels equivalent to the PT thresholds using the measured TcB plus 50 μmol/L and thus correcting for the reported underestimation of the TSB level by the JM-103, 3.) TcB cut-off levels at 70% of the PT thresholds considering the large variation in differences between TcB and TSB, and 4.) a combination of formulas 2 and 3: TcB cut off levels are similar to TcB levels plus 50 μmol/L at 70% of PT thresholds.

We determined sensitivity, TNs, and FNs, except for a birth weight of > 2000 g due to the relatively small number of cases in this category. Sensitivity is the proportion of infants with a TSB level higher than the threshold correctly identified with a TcB cut-off value higher than the TSB threshold. Mathematically, this can be expressed as true positive (TP) TcB measurements divided by the TP plus FN TcB measurements, i.e. the total number of infants with a TSB > threshold multiplied by 100%.

The true negative (TN) measurements are the number of TcB measurements lower than the PT threshold in the presence of corresponding TSB levels that are also lower than this threshold. The number of blood samples that would have been reduced is represented by the TN TcB measurements.

False negative TcB measurements are TcB levels that inadvertently missed high TSB levels that require treatment. The FN TcB measurements were identified as the number of TcB levels below the TSB PT threshold whereas the actual corresponding TSB levels were above this threshold. The percentage FN was calculated as percentage of all measurements: ((FN/TN+FN+TP+FP) x 100).

In addition, other accuracy parameters, such as specificity, positive predictive value (PPV), negative predictive value (NPV), and positive and negative likelihood (LH) ratios, were calculated in order to compare the different cut-off methods. Finally, a receiver operating characteristic (ROC) curve was constructed with overall data of all four TcB cut-off methods. Microsoft Office Excel 2010 (Microsoft Corporation, Redmond, WA, USA) and IBM SPSS for Windows, Version 23.0 (IBM Corporation, Armonk, NY, USA) were used for data entry and analysis. All relevant data are in S1 File.

## Results

**Fig 1:**
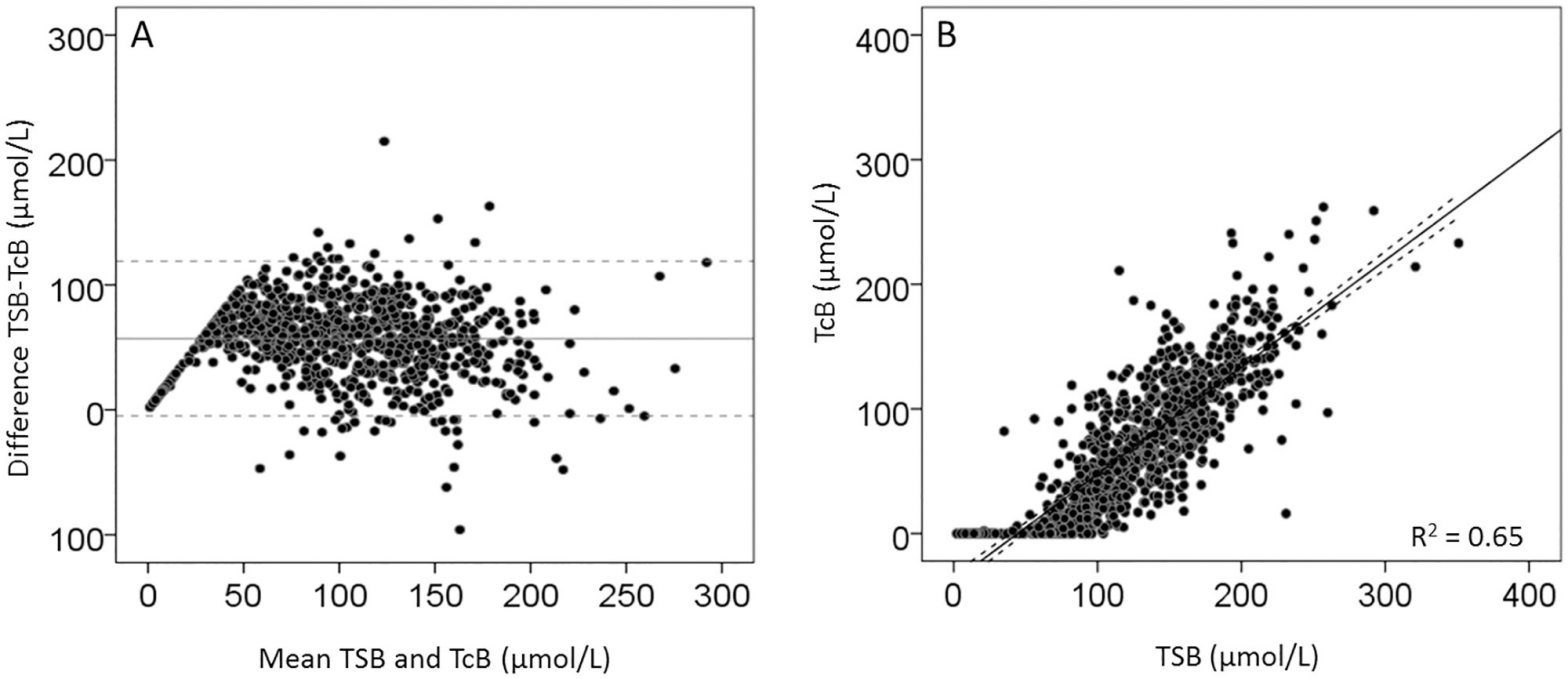
Bland Altman and correlation plots for all TcB and TSB measurements. Fig 1A shows the agreement between TSB and TcB and Fig 1B shows the correlation between TSB and TcB. The x–axis of Fig 1A shows the mean of TSB and TcB in μmol/L and the y–axis shows the difference between TSB and TcB (μmol/L). The horizontal line in Fig 1A represents the mean difference: 57 μmol/L, the dotted lines represent the 95% confidence intervals:—5 to 119 μmol/L. 17.1μmol/L = 1 mg/dL bilirubin.

Out of the 114 patients included in the BARTrial 109 infants were included in this analysis. TcB measurements were not performed in four infants (three for unknown reasons and one infant died on the first day after birth). For one infant the timing of the TcB measurements was not documented. The majority of the infants, ie, 96 (88%) were Caucasian and 66 (61%) were boys. The mean (±SD) GA was 29.4 ± 1.6 weeks and the mean (±SD) birth weight was 1282 g ± 316 g. A total of 98 infants (91%) received PT with a mean (SD) total duration of 68 (±52) hours. Eleven infants (9%) did not receive any PT during the study period. A total of 856 paired TSB and TcB levels were obtained, with a mean of 8, ranging from 1 to 14 paired measurements per patient. The postnatal course of TSB and TcB were comparable with peak levels around Day 3. Fig 1 shows the agreement and correlation between TSB and TcB levels for all 856 measurements.

**Fig 2:**
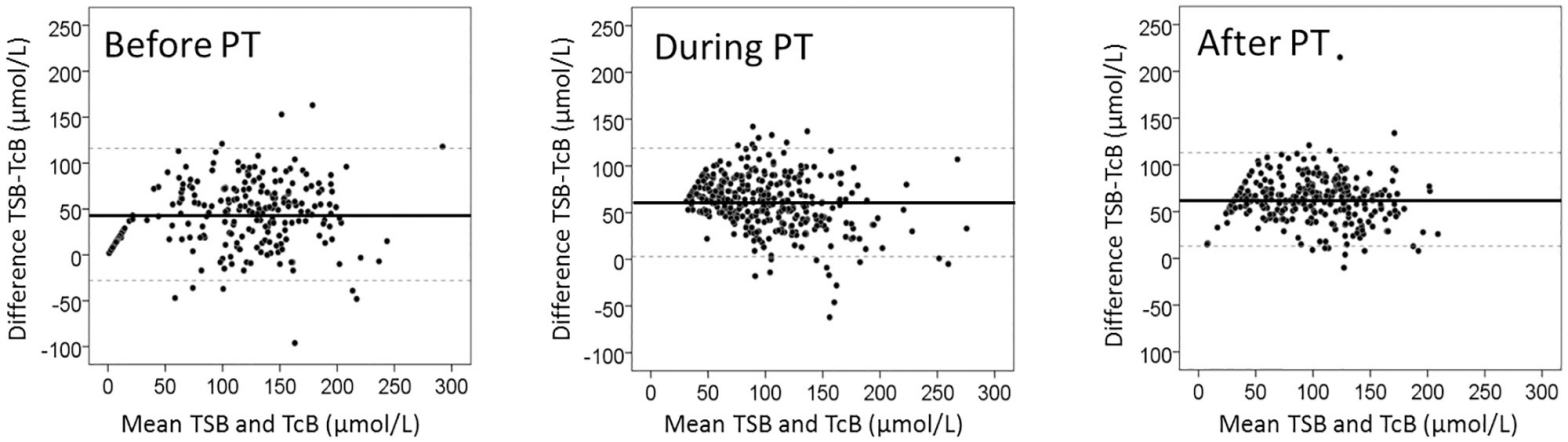
Bland Altman plots before, during, and after phototherapy. The agreement between TSB and TcB before, during, and after PT. The x–axis shows the mean of TSB and TcB (μmol/L) and the y–axis shows the difference between TSB and TcB (μmol/L). The horizontal line represents the mean difference; the dotted lines represent the 95% confidence interval. 17.1μmol/L = 1 mg/dL bilirubin.

Almost all (96%) TcB levels except 35 (4%) underestimated the TSB level with a mean (±SD) of 57 ± 31 μmol/L. The overall correlation between TSB and TcB before, during, and after PT was similar (R = 0.81, 0.81, and 0.84, respectively) and statistically significant (*P* < 0.01). Fig 2 shows the agreement between TSB and TcB before, during, and after PT. Table 1 and Fig 2 show that the mean difference between TSB and TcB was significantly lower (*P* < 0.05) before PT (44 ± 36 μmol/L) than during (61 ± 29 μmol/L), and after PT (63 ± 25 μmol/L).

**Table 1:** Total serum bilirubin and transcutaneous bilirubin levels.

	TSB (μmol/L)	TcB (μmol/L)	Mean difference (TSB–TcB) (μmol/L)
**Total (n = 856)**	134 ± 48	77 ± 51	57 ± 31
**Before PT (n = 229)**	139 ± 61	95 ± 56	44 ± 36
**During PT (n = 335)**	133 ± 42	72 ± 50	61 ± 29*
**After PT (n = 292)**	131 ± 43	68 ± 44	63 ± 25*

PT, phototherapy; TSB, total serum bilirubin; TcB, transcutaneous bilirubin, 17.1 μmol/L = 1 mg/dL bilirubin. Data are represented as mean (±SD).

* *P* < 0.05 before PT versus during and after PT.

**Fig 3:**
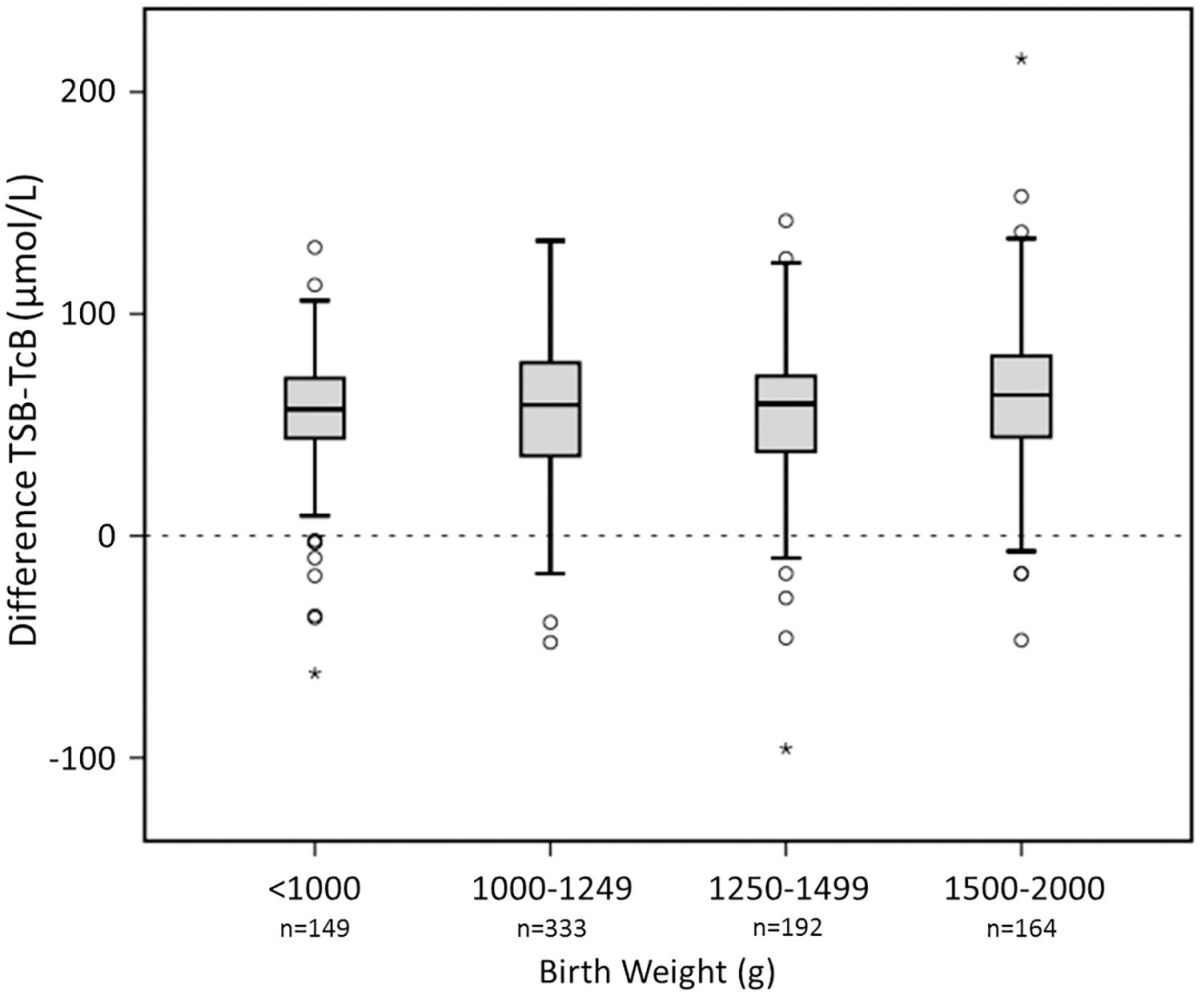
Birth weight categories and the mean difference between TSB and TcB. The y–axis displays the mean difference between TSB and TcB in μmol/L. TSB, total serum bilirubin in μmol/L; TcB, transcutaneous bilirubin in μmol/L; 17.1μmol/L = 1 mg/dL bilirubin. The median is marked by the horizontal line in the central box. The boxes are limited by the 25th and 75th percentiles. The whiskers (┴) represent the minimum and maximum TSB and TcB levels. Outliers (○) and extremes (star symbol) are depicted separately. The dashed line is the line at origin.

Infants weighing more than 2000 g had the highest mean TSB levels (228 ± 58 μmol/L) compared to 171 ± 38 μmol/L, 147 ± 40 μmol/L, 120 ± 30 μmol/L, and 97 ± 34 μmol/L for infants of 1500–2000 g, 1250–1499 g, 1000–1249 g, and < 1000 g, respectively; *P* < 0.001). Fig 3 shows that the mean difference between TSB and TcB levels was similar for all the studied birth weight categories.

**Fig 4:**
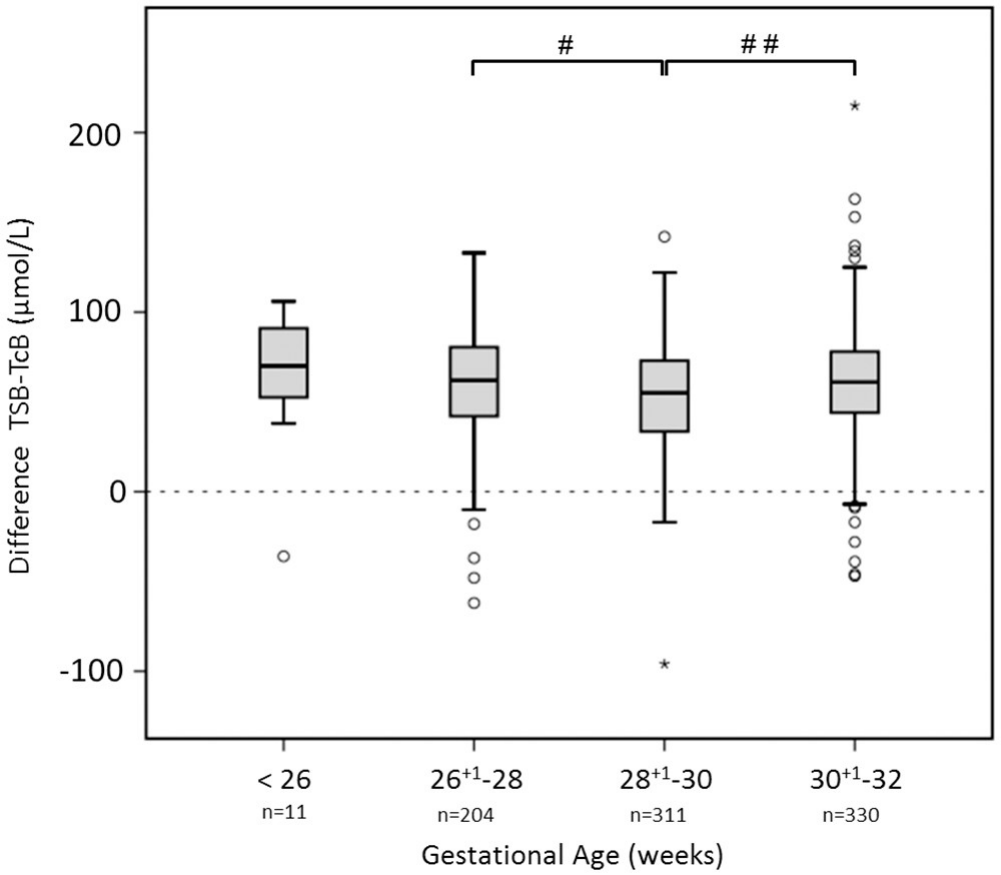
Gestational age categories and the mean difference between TSB and TcB. The y–axis displays the mean difference between TSB and TcB in μmol/L. TSB, total serum bilirubin in μmol/L; TcB, transcutaneous bilirubin in μmol/L; 17.1μmol/L = 1 mg/dL bilirubin. The median is marked by the horizontal line in the central box. The boxes are limited by the 25th and 75th percentiles. The whiskers (┴) represent the minimum and maximum TSB and TcB levels. Outliers (○) and extremes (star symbol) are depicted separately. The dashed line is the line at origin. Significant differences between groups are marked in the top of Fig 4 by # (*P* < 0.05) or ## (*P* < 0.01), respectively.

It appeared that mean differences between TcB and TSB were affected by GA. Fig 4 shows that the mean difference of TSB and TcB for infants with a GA of 28 + 1–30 weeks (52 ± 30 μmol/L) was significantly lower than the mean difference of infants of 26 +1–28 weeks (59 ± 32 μmol/L, *P* < 0.05), and 30 + 1–32 weeks (61 ± 30 μmol/L, *P* < 0.01).

The length of time after stopping PT did not appear to have a statistically significant effect on the mean differences between TSB and TcB. Mean differences between TSB and TcB levels were 60 ± 22 μmol/L, 66 ± 19 μmol/L, and 64 ± 20μmol/L, respectively at 12 hours (n = 54), 24 hours (n = 47), and > 48 hours (n = 101) after PT was stopped. Table 2 shows the percentages of blood samples that would be reduced in all birth weight categories and corresponding false negative rate percentages for the different formulas to determine TcB cut-off levels. For these calculations we used 724 paired TcB and TSB measurements, ie, all paired data after 48 postnatal hours. When TcB cut-off levels equivalent to PT thresholds were used, the mean number of blood samples that would be reduced in all birth weight categories was 81%, with a mean FN rate of 16%, up to 20% in infants weighing < 1250 g. The mean percentage of blood samples that would be reduced changed to 75%, with 9% FN TcB measurements, after adding 50 μmol/L to the measured TcB levels (to correct for any underestimation). TcB cut-off levels at 70% of the TSB thresholds showed similar results. Combining TcB + 50 μmol/L and 70% of the TSB PT threshold resulted in a very low FN rate of 2% and a 42% reduction in the need for blood sampling.

**Table 2:** Effect of different formulas for optimal TcB cut-off levels on blood sample reduction and on false negative percentages per birth weight group.

**Applied TcB cut-off level**			**TcB > TSB**		**TcB + 50 > TSB**	
**Birth weight (g)**	n = 724	TSB threshold (μM)	Reduction in blood samples (%)	FN (%)	Reduction in blood samples (%)	FN (%)
**≤ 999**	125	100	73	20	64	12
**1000–1249**	297	150	78	20	71	10
**1250–1499**	168	190	88	11	82	5
**1500–1999**	134	220	89	9	83	6
**Overall**			81	16	75	9
**Applied TcB cut-off level**			**TcB > 70% TSB**		**TcB + 50 > 70%TSB**	
**Birth weight (g)**	n = 724	70% TSB threshold (μM)	Reduction in blood samples (%)	FN (%)	Reduction in blood samples (%)	FN (%)
**≤ 999**	125	70	69	14	40	4
**1000–1249**	297	105	73	11	44	2
**1250–1499**	168	133	79	5	42	0
**1500–1999**	134	154	81	5	40	2
**Overall**			75	9	42	2

The percentage of blood samples that would have been reduced corresponds to the TN TcB measurements (i.e. TcB and TSB lower than treatment threshold), divided by total measurements and multiplied by 100. The FN percentage was calculated from the number of TcB levels below the PT threshold, whereas actual TSB levels were above this PT threshold, divided by the total number of TcB measurements and multiplied by 100. N, number of measurements in the corresponding birth weight category; TSB, total serum bilirubin (17.1 μmol/L = 1 mg/dL bilirubin); TcB, transcutaneous bilirubin; FN, false negative TcB measurement.

Table 3 shows that sensitivity, and NPVare highest and negative LH is lowest using the latter cut-off level, whereas specificity, PPV, and positive LH are lowest with this particular level. S1 Fig shows the ROC curve with overall sensitivity and specificity data of the four methods to define TcB cut-off levels.

**Table 3:** Accuracy parameters of different TCB cut-off levels.

**Applied TcB cut-off level: TcB > TSB**								
Birth weight (g)	n = 724	**TSB threshold (μM)**	**Sens**	**Spec**	**NPV**	**PPV**	**LH neg**	**LH pos**
≤ 999	125	100	26	100	78	100	0.74	∞
1000–1249	297	150	6	99	80	67	0.94	7.58
1250–1499	168	190	10	100	89	100	0.90	∞
1500–1999	134	220	20	100	91	100	0.80	∞
Overall			14	99.7	84	90	0.87	40.36
**Applied TcB cut-off level: TcB +50 > TSB**								
**Birth weight (g)**	n = 724	**TSB threshold (μM)**	**Sens**	**Spec**	**NPV**	**PPV**	**LH neg**	**LH pos**
≤ 999	125	100	56	88	84	63	0.50	4.62
1000–1249	297	150	52	90	88	57	0.54	5.05
1250–1499	168	190	57	94	94	57	0.46	9.33
1500–1999	134	220	47	93	93	47	0.57	6.94
Overall			53	91	90	57	0.51	6.04
**Applied TcB cut-off level: TcB > 70% TSB**								
Birth weight (g)	n = 724	**70% TSB threshold (μM)**	**Sens**	**Spec**	**NPV**	**PPV**	**LH neg**	**LH pos**
≤ 999	125	70	50	95	83	77	0.53	9.10
1000–1249	297	105	47	92	87	62	0.58	6.11
1250–1499	168	133	62	90	94	48	0.42	6,50
1500–1999	134	154	60	91	95	48	0.42	6.49
Overall			52	92	89	59	0.53	6.35
**Applied TcB cut-off level: TcB+50 > 70% TSB**								
Birth weight (g)	n = 724	**70% TSB threshold (μM)**	**Sens**	**Spec**	**NPV**	**PPV**	**LH neg**	**LH pos**
≤ 999	125	70	85	55	91	41	0.27	1.89
1000–1249	297	105	92	56	96	36	0.14	2.10
1250–1499	168	133	100	48	100	21	0.00	1.91
1500–1999	134	154	80	45	95	16	0.44	1.46
Overall			90	52	96	29	0.19	1.87

Sens, sensitivity; Spec, specificity; NPV, negative predictive value; PPV, positive predictive value; LH neg, negative likelihood ratio; LH pos, positive likelihood ratio.

## Discussion

In the present study we found that TcB levels measured on the covered hipbone showed a strong correlation and good agreement with TSB levels in preterm infants of less than 32 weeks’ GA before, during, and after PT. The data also showed that TcB persistently underestimated the TSB level and that PT did affect this underestimation: the underestimation increased from approximately 45 μmol/L to approximately 60 μmol/L and this effect remained for as long as 48 hours after PT was stopped. We present a formula to correct this underestimation and the large variation in differences between TSB and TcB levels. It is possible to reduce blood sampling by 40%, with a minimal risk of missing preterm infants of less than 32 weeks’ GA with significant hyperbilirubinemia, by applying the following formula: add 50 μmol/L to the measured TcB value and use 70% of the TSB PT treatment threshold.

Our data on the underestimation of TSB by TcB measurements are in line with previous studies in preterm infants with TcB measurements taken on the forehead, the sternum, or on the abdomen [3,17,24]. This is the first study in preterm infants to report on TcB measurements that were obtained on the hipbone. The persistent underestimation of TSB by the JM–103 instrument of up to 50 μmol/L therefore seems to occur irrespective of the measurement site. In the present study we found that during PT the underestimation of TSB was higher by approximately 15 μmol/L and remained higher after stopping PT. In preterm infants, the hipbone is covered by a diaper and thus shielded from most of the PT light to avoid bleaching of the skin. Hipbone TcB measurements preclude the need for glued patches PT as were used by other researchers in preterm infants. Zecca and colleagues compared TSB levels with TcB levels measured with the BiliCheck on the covered versus the uncovered skin on the forehead during PT [9]. They found a statistically significant difference between TSB minus TcB levels: 3.4 μmol/L on the covered skin versus 55 μmol/L for measurements on the uncovered skin after 52 hours of PT. Nanjundaswamy and colleagues used a comparable methodology with a standard PT eye patch on the forehead to compare TSB with TcB levels in preterm infants [10]: The correlation between TSB and TcB levels was significantly weaker for TcB measurements on the uncovered skin compared to TcB measurements on the covered skin, and compared to the control measurements before PT. By analogy, Knupfer and colleagues found a higher correlation between TSB and TcB levels for infants without PT compared to infants with PT [11]. The lower correlation between TSB and TcB measurements on the covered skin during PT could be due to some PT light leakage along the edges of the photo-opaque patch. We found no effect of PT on the correlation between TSB and TcB. We did, however, find an effect of PT on the agreement of both measurements: underestimation of TSB levels increased during PT. This might be explained by some PT light transmitting to the skin under the diaper. The irradiance level under the diaper of infants on a Bilibanket (with an irradiance of 17 μW/cm^2^/nm) was only 15% (2.6 μW/cm^2^/nm–personal observation by DEVvI). Nevertheless, this does not explain the persistent underestimation after PT was stopped. This is, at least in part, in line with previous data of TcB during and after PT in fullterm infants. Fonseca and colleagues described persistent underestimation of TSB by TcB measurements taken on skin exposed to PT, but not on covered skin, whereas Casnoch and colleagues found persistent underestimation after PT on all sites, including the covered lower abdomen (with the lowest TSB-TcB difference) [25,26]. We speculate that the effect of PT on TcB measurements is related to bilirubin kinetics in the skin and subcutaneous tissue. PT exerts its overall hypobilirubinemic effect mainly on intravascular bilirubin, and it bleaches the skin. Apparently this bleaching occurs faster than TSB reduction and persists after PT is stopped. The underestimation of TSB increases with approximately 15–20 μmol/L after PT is started and thus seems of modest clinical relevance. There is a rather large variation of this underestimation, irrespective of birthweight and gestational age.

We determined the effects of previously published formulas for TcB cut-off levels that aim to reduce the need for blood sampling without missing high TSB levels. At first we compared TcB directly to TSB concentrations and found an 81% reduction in the need for blood sampling, but with an unacceptable high percentage of FNs (16%). Subsequently, we corrected for the underestimation by adding 50 μmol/L to the measured TcB level and still noticed a marked reduction (75%) in the need for blood sampling, but a substantial risk (9%) of missing significant hyperbilirubinemia. Similar results were obtained when we corrected for the large variation in differences between TSB and TcB levels by lowering the PT threshold levels to 70% of the original PT threshold. The combined cut-off method of TcB+50 and 70% of the PT threshold resulted in a smaller reduction (42%) of blood samples with the lowest FN rate of 2%, and a high negative predictive value (Tables 2 and 3). For preterm infants with imminent bilirubin neurotoxicity it is obvious to apply TcB cut-off levels that minimize the risk of missing high TSB levels. The low FN rate and high NPV of all cut-off levels could be explained by the cephalocaudal progression of jaundice: if TcB measured on the hipbone is low, a high TSB is rare. Our data on the number of blood samples that may be reduced and the FN rates are in line with previous data (up to 40% reduction and no FNs) [10,12,27,28]. Nevertheless, any comparison between previous studies is hazardous. Many factors influence TcB measurements: GA, postnatal age, skin color/ethnicity, measurement site, TcB devices and its algorithm, the laboratory method for TSB measurement, and the local treatment thresholds.

This study was performed with the JM-103 instrument that has been on the market for a long time and is still used frequently. More importantly, the basic functionality of the successor to the JM-103, the JM-105, is similar in so far as its measuring probe, hardware, and software are identical [29]. Therefore we expect that our findings can be extrapolated to TcB measurements with the newer instrument.

We acknowledge a few limitations to our study. Firstly, our results, especially the numerical values, cannot be generalized to the entire preterm infant population, because the majority of our study population is Caucasian and TcB measurements may vary in children of different skin color. Secondly, the TcB cut-off rule with the lowest chance to inadvertently miss high TSB levels most likely depends on the technical specifications of the TcB instrument, which are brand-specific. Therefore this optimal cut-off rule may not be applicable to TcB instruments of other brands. Finally, alternative guidelines will impact our results; the consequence of a specific cut-off level depends on the treatment threshold utilized.

The strengths of our study include the measurement of a skin area protected from PT light by a diaper, rather than needing to cover a patch of skin. The hip bone area is easy for nurses to access and this also applies to preterm infants on continuous positive airways pressure support with equipment covering their heads, and may be used with any TcB device without additional patches. Thus far, only one study on fullterm infants reported on TcB measurements on the lower abdomen covered by a diaper [26]. However, although the abdomen and hip bone are both in Kramer Zone 3, abdominal TcB measurements lack a firm underground and thus may yield different TcB levels.

Additionally, we present a prospective series of paired TcB-TSB measurements before, during, and after PT in preterm infants with different GA ranges and birth weights that adds to the generizability of our findings. Finally, we presented Bland Altman plots that do not depend on treatment thresholds and are more useful than correlations in clinical practice.

Based on our results and in line with previous data and recommendations [9,30,31], we recommend using TcB measurements on the hip bone covered by a diaper in preterm infants without, but also in infants undergoing PT. The latter TcB measurements provide a non-invasive means to estimate TSB during treatment and may individualize timing of TSB sampling: Using TcB during PT may result in earlier or later measurement of TSB than the customarily TSB measurements after 12 or 24 hours and may aid decision-making regarding PT duration. We advise to be aware of changes in the algorithm of the TcB device that might influence TSB estimation. Therefore we recommend checking the accuracy of the device regularly by performing paired TSB and TcB measurements.

## Conclusion

PT enhances underestimation of TSB by TcB that lasts after PT is stopped when measured on the covered hip bone in preterm infants of 32 weeks’ GA or less. A reduction of approximately 40% of blood samples is feasible without a substantial risk of missing high TSB levels, using a TcB+50 μmol/L cut-off level at 70% of the PT treatment threshold.

## Supporting information

S1 Table Contains the items of the Standards for Reporting of Diagnostic Accuracy (STARD) checklist, enabling to reporting studies of diagnostic accuracy according to a standard method. (DOCX)

S1 Fig Contains a receiver operating characteristic (ROC) curve with overall data of four methods to define TcB cut-off levels. (TIF)

S1 File Contains all relevant data. (SAV)
