# Supplementary figures and images for: Should transcutaneous bilirubin be measured in preterm infants receiving phototherapy? The relationship between transcutaneous and total serum bilirubin in preterm infants with and without phototherapy
Source: PLoS One. 2019 Jun 14;14(6):e0218131. doi: 10.1371/journal.pone.0218131 (PMC6568417; doi:10.1371/journal.pone.0218131)

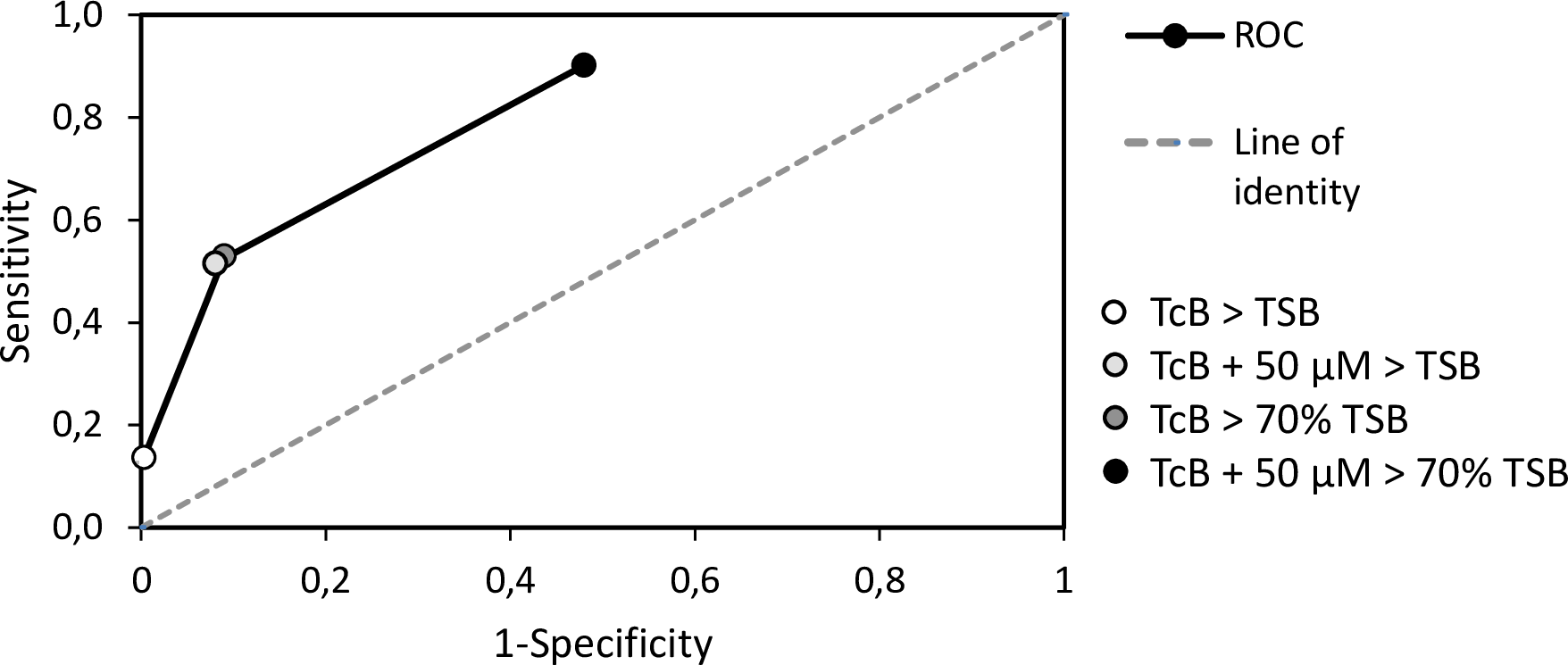

Supplement: S1 Fig — (TIF) [file pone.0218131.s002.tif]
